# Evolution of the notochord

**DOI:** 10.1186/s13227-015-0025-3

**Published:** 2015-10-05

**Authors:** Giovanni Annona, Nicholas D. Holland, Salvatore D’Aniello

**Affiliations:** Department of Biology and Evolution of Marine Organisms, Stazione Zoologica Anton Dohrn, Villa Comunale, 80121 Naples, Italy; Marine Biology Research Division, Scripps Institution of Oceanography, University of California at San Diego, La Jolla, CA 92093 USA

**Keywords:** Notochord, Stomochord, Pygochord, Axochord, Annelid scenario, Enteropneust scenario

## Abstract

A notochord is characteristic of developing chordates (which comprise amphioxus, tunicates and vertebrates), and, more arguably, is also found in some other animals. Although notochords have been well reviewed from a developmental genetic point of view, there has heretofore been no adequate survey of the dozen or so scenarios accounting for their evolutionary origin. Advances in molecular phylogenetics and developmental genetics have, on the one hand, failed to support many of these ideas (although, it is not impossible that some of these rejects may yet, at least in part, return to favor). On the other hand, current molecular approaches have actually stimulated the revival of two of the old proposals: first that the notochord is a novelty that arose in the chordates, and second that it is derived from a homologous structure, the axochord, that was present in annelid-like ancestors. In the long term, choosing whether the notochord is a chordate novelty or a legacy from an ancient annelid (or perhaps an evolutionary derivative from precursors yet to be proposed) will probably require descriptions of gene regulatory networks involved in the development of notochords and notochord-like structures in a wide spectrum of animals. For now, one-way forward will be studies of all aspects of the biology of enteropneust hemichordates, a group widely thought to be the key to understanding the evolutionary origin of the chordates.

## Background

**Fig. 1:**
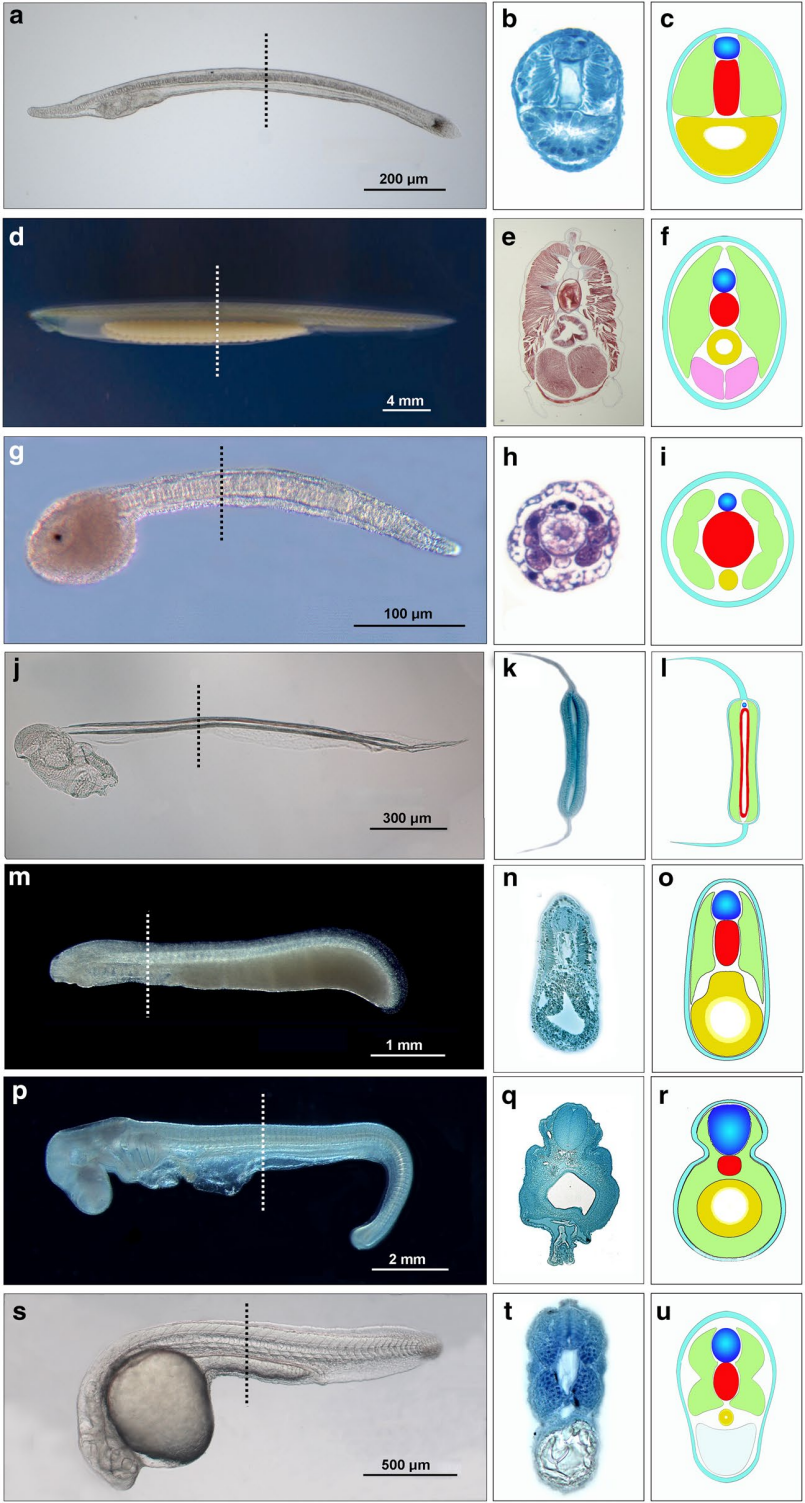
Chordate notochords in side views, cross sections (through the dotted line in each side view), and diagrams of the cross sections. Structures shown in the diagrams include the nerve cord (*blue*), notochord (*red*), axial musculature (*green*), endoderm (*yellow*), and gonads (*purple*). **a**–**f** Amphioxus, *Branchiostoma lanceolatum*, **a**–**c** 3-day larva and **d**–**f** adult. Tunicates: **g**–**i** late tailbud larva of an ascidian, *Ciona intestinalis,* and **j**–**l** an adult appendicularian, *Oikopleura dioica*. **m**–**o** Stage 26 larval lamprey, *Petromyzon marinus*. **p**–**r** Stage 24 embryonic shark, *Scyliorhinus canicula.* **s**–**u**) Pharyngula stage embryo of teleost, *Danio rerio*

Animals swimming by undulation include some vertebrates (especially elongate fishes [1]) and diverse invertebrates [2–13] (Table 1). These movements are generated when longitudinal muscles contract against a hydrostatic skeleton. The most typical hydrostatic skeleton among invertebrates results from the constraint of internal fluids and soft tissues by a rigid or elastic body wall. A second kind of hydrostatic skeleton is an internal rod, the notochord, which functions as a flexible compression strut [14]. A notochord is present in the phylum Chordata (comprising three subphyla: amphioxus, tunicates, and vertebrates) and, more arguably, in some other animals. Among invertebrate chordates (Fig. 1a–l), amphioxus and appendicularian tunicates retain the notochord through the adult stage, while ascidian tunicates typically possess it only in the larval period. In the vertebrates (Fig. 1m–u), the structure is always present during early developmental stages, but, with a few exceptions (e.g., hagfishes, lampreys, and sturgeons), it is largely replaced in adults by an externally added spinal column of cartilage or bone [15]. The mature spinal column sometimes continues to function for undulatory locomotion.

**Table 1:** Invertebrates that swim by undulating the entire body or its posterior region

Phylum	References
Ctenophora (escape response of adult Venus’ girdle)	[2]
Platyhelminthes (cercaria larvae of trematodes)	[3]
Chaetognatha (adults of planktonic species)	[4]
Nematoda (adults of some species)	[5]
Nematomorpha (only in *Nectonema* spp.)	[6]
Arthropods (mayfly larvae)	[7]
Annelida (adults)	
Polychaetes (in a few species)	[8]
Oligochaetes (in a few species)	[9]
Leeches (in numerous species)	[10]
Hemichordata (known only for adults of one species)	[11]
Chordata	
Cephalochordata (larval and adult amphioxus)	[12]
Tunicata (ascidian larvae, appendicularian adults)	[13]

The chordate notochord runs along almost the entire rostrocaudal body axis of amphioxus, but terminates anteriorly in the region of the hindbrain of tunicates and vertebrates. Developmental genetic aspects of chordate notochords have recently been thoroughly reviewed [16, 17], and only the most salient features will be summarized in the present text and in Table 2, which also compares the morphology of notochords for amphioxus, tunicates, and vertebrates [18–24]. Because there are some cytological differences in the notochord among the three major chordate groups, its homology has sometimes been questioned [25]. In addition, the germ layer source of notochords has also been controversial, first because they often originate in embryonic regions where the germ layers are not clearly delineated [26] and second because of confusion over the distribution of the nascent mesoderm in amphioxus gastrulae [27]. From currently available data [28], we will assume here that all chordate notochords are homologous in spite of differences in cytological detail and that all arise from mesendoderm.

**Table 2:** Salient features of notochords compared among amphioxus, tunicates, and vertebrates

	Amphioxus larvae and adults	Tunicate larvae	Vertebrate embryos^a^
Cell types	I. Discoidal cells (stacked like coins^b^; each cell containing transverse myofilaments) II. Müller cells: sparsely distributed; no known function	Early larva: discoidal cells (stacked like coins^b^; no myofilaments) Late larva: above cells change to squamous epithelium around fluid-filled lumen^c^	I. Inner core cells^d^: each with a large vacuole; no myofilaments II. Surrounding epithelial cell layer
Extracellular sheath	Inside to out: external lamina^e^, circular collagen layer, and longitudinal collagen layer^f^	External lamina^e^	Inside to out: external lamina^e^, circular collagen layer, and longitudinal collagen layer^f^
Organizer genes involved in notochord formation^g^	Comparable to those of vertebrates	Highly divergent^h^	Comparable to those of amphioxus
Hedgehog from notochord involved in patterning central nervous system	Yes^i^	No^j^	Yes

^a^Also in adults of more basal vertebrates (e.g., cyclostomes, sturgeons)

^b^During notochord formation, this arrangement is attained rapidly by inconspicuous cell movements [18, 78]; the more marked cell migrations and intercalations establishing the vertebrate notochord have been termed convergent extension [19]

^c^Transitional stages described for ascidians in [18]; there is a similar arrangement in adult appendicularian tunicates [20]

^d^Often considered a kind of cartilage [15]; the inner core cells are embedded in an extracellular matrix that is disconcertingly scanty for cartilage, although including some macromolecules characteristic of that tissue [21]

^e^Also sometimes termed the basal lamina or the elastica interna

^f^Also sometimes termed the elastica externa

^g^Discussed in [22]

^h^Discussed in [113]

^i^Discussed in [23]

^j^Discussed in [24]

There have previously been few reviews covering ideas about notochord evolution. The most extensive of these [29] covered less than half of the scenarios published before 1940 and ended by firmly dismissing (in the event, prematurely) any possibility that chordate notochords might be a legacy from arthropods or annelids. Therefore, our first purpose here is to summarize the scattered literature on this subject, which spans a century and a half. Notochord evolution has often been discussed within the context of scenarios for the invertebrate-to-vertebrate transition [30] (which are traditionally named after a key invertebrate group perceived as ancestral to vertebrates). At present, many of these old ideas have lost their appeal due to progress in molecular phylogeny and developmental genetics. As a caveat, however, science is not invariably a story of constant progress that renders past work of little consequence [31]. There is always the chance that some features of the currently ignored scenarios will be revived in the light of modern discoveries. At present, however, only two of the old theories look attractive in the light of molecular genetics and have become the subject of active research programs—the first proposes that the vertebrate notochord is a legacy from non-chordate invertebrates, and the second considers that the structure was invented de novo within the chordates. Our second purpose is to examine the modern evidence that has been invoked to support these two contending points of view.

## Review

### Notochords in pre-Darwinian times

The notochord was discovered in 1828 in chick embryos by von Baer [32], who called it sometimes the *dorsal strand* (*Rückensaite*) and sometimes the *chorda dorsalis*. The latter term predominated during the nineteenth century, but, for convenience, we will refer to the structure simply as the *notochord*. Following von Baer’s discovery, notochords were soon found in embryos of other vertebrates [33, 34] as well as in adults of amphioxus (classified then as fishes) [35]. Consequently, the homology of such structures among all vertebrates came to be widely accepted. Such a homology, being restricted to vertebrates, was agreeable to von Baer, who was willing to consider limited transformation within each of Cuvier’s four embranchements—namely, the vertebrates, radiates (cnidarians and echinoderms), articulates (arthropods and annelids), and molluscs [36]. In contrast, he firmly denied any evolutionary relationship between one embranchement and the next.

### Initial evolutionary ideas about notochords

The 1859 publication of Darwin’s *Origin of Species* triggered numerous scenarios for the invertebrate-to-vertebrate transition [30], most including speculations on the evolutionary origin of the notochord. It was not long before von Baer’s opposition to cross embranchement homologies was widely challenged, most irritatingly by a student in his own department at Saint Petersburg University—Alexander Kowalevsky. The seeds of the conflict were sown in 1861 when Kowalevsky visited Heidelberg University and met Arnold Pagenstecher, who had recently described advanced larvae of amphioxus netted from the North Sea plankton [37]. In the opinion of Vucinich [38], Pagenstecher convinced Kowalevsky to go to Naples, Italy, to study the early embryology of amphioxus, although definitive evidence for that is lacking. In any event, Kowalevsky visited Naples from late 1863 through much of 1864 to work on the embryology of several invertebrates, most importantly amphioxus and ascidian tunicates.

**Fig. 2:**
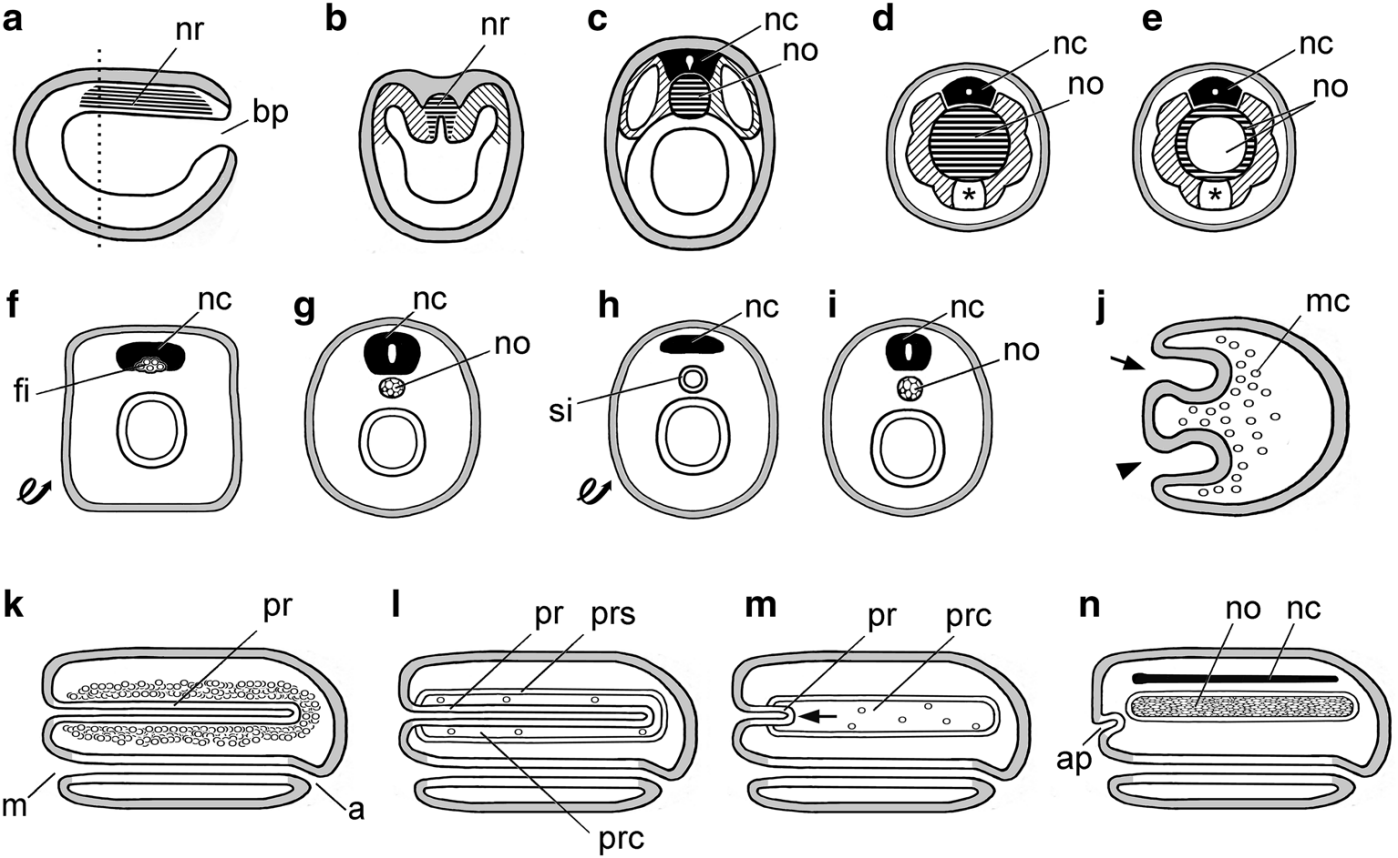
Notochords in phylogeny: invertebrate chordates, annelids, and nemerteans. **a** Midsagittal section of early neurula of amphioxus with blastopore (bp) and notochord rudiment (nr, horizontal hatching). **b** Cross section at level indicated by *dotted line* in **a**; notochord rudiment (nr) and somites indicated, respectively, by horizontal and diagonal hatching. **c** Cross section of later neurula of amphioxus showing notochord (no) and dorsal nerve cord (nc); somites indicated by diagonal hatching. **d**, **e** Early and late larvae, respectively, of an ascidian tunicate. Cross sections through the tail showing nerve cord (nc); muscles (diagonally hatched) and notochord (no); *asterisk* indicates endodermal strand (discovered by Seeliger [114]). **f**, **g** Inverted annelid scenario (after Semper [48]); following inversion (**f** *looped arrow*), fibers (fi) associated with the nerve cord (nc) are precursors of the notochord (no) in **g**. **h**, **i** Variant annelid theory (after Ehlers [50]); annelid after inversion (**h**, *looped arrow*), the position of the siphon (si) corresponds to the vertebrate notochord (no) in **i**. **j**–**n** Nemertean scenario (after Hubrecht [54]); gastrula (**j**) has a first invagination (*arrowhead*) for gut and a second invagination (*arrow*) for the proboscis, while mesenchyme cells (mc) ingress into the blastocoel. Subsequently **k**, a through gut forms from mouth (m) to anus (a), and mesenchyme cells condense around the proboscis (pr). **l** Schizocoely produces a proboscis coelom (prc) and a proboscis sheath (prs). The *arrow* in **m** indicates the proboscis (pr) pulling out of the proboscis coelom (prc), leaving behind a few mesenchyme cells. In **n**, the mesenchyme cells in the proboscis coelom have extensively proliferated to form the notochord (no); the remains of the proboscis have become the anterior pituitary (ap), while the dorsal nerve cord (nc) has formed by the dorsal migration and fusion of the lateral nerve cords

When Kowalevsky published on the embryology of amphioxus [39], he included a description of the early development of the notochord. He mistakenly thought the structure originated from segmental muscle cells, but corrected himself later [40] by finding that it arose from the mid-dorsal roof of the archenteron (Fig. 2a–c). He also erred initially in claiming that the newly formed notochord comprised a population of individual cells that soon merged into a syncytium [39]. This is surprising because the correct answer had already been published [37]: namely, the amphioxus notochord consists chiefly of a row of persistently separate discoidal cells organized like a stack of coins. Importantly, Kowalevsky demonstrated that the early development of amphioxus is invertebrate-like, but the later embryology is vertebrate-like; however, he did not immediately express his opinion about the evolutionary implications of his discovery, perhaps to avoid antagonizing von Baer. Even so, Metchnikoff [41] was representative of many others in jumping to the obvious conclusion that “the major features of amphioxus development are intermediate between the development of vertebrates and that of lower animals” (our translation).

Kowalevsky’s research on amphioxus (excepting the mistakes noted above) was superb, but it was his publication on the embryology of ascidian tunicates [42] that brought him international fame. He followed the ascidian development through pre-metamorphic larvae, in which he discovered a notochord. Although vague about the cellular sources of the structure (at the time, nothing was known of embryonic cell lineages), he clearly described its early appearance as a solid cord of cells (Fig. 2d) as well as its further transition into a cellular sheath surrounding a fluid-filled lumen (Fig. 2e). The fully developed, coelom-like notochord in ascidians influenced some subsequent ideas about notochord evolution, as will be discussed further below.

The most consequential feature in Kowalevsky [42] was his proposal that ascidians were closely related to vertebrates. This was a clear challenge to von Baer, who considered ascidians to be molluscs related to shipworms largely on the basis of the incorrect homology he made between the ciliated gill slits of (adult) ascidians and those of bivalves. Kowalevsky’s evolutionary conclusions were particularly irksome to von Baer, first for violating the genetic purity of the embranchements and second for attracting the attention of Darwin [43], who wrote that “…Ascidians are related to the Vertebrata, in their manner of development, in the relative position of the nervous system, and in possessing a structure closely like the *chorda dorsalis* of vertebrate animals. …We should thus be justified in believing that at an extremely remote period a group of animals existed, resembling in many respects the larvae of our present Ascidians, which diverged into two great branches—the one retrograding in development and producing the present class of Ascidians, the other rising to the crown and summit of the animal kingdom by giving birth to the Vertebrata.” Due to this high-profile publicity, Kowalevsky’s ascidian work quickly became the target of vigorous criticisms, but he countered them all successfully over the next few years, as thoroughly reviewed by Beeson [44].

### The original annelid scenario

Because of Kowalevsky’s embryological work, most biologists soon came to regard tunicates no longer as molluscs, but as close relatives of vertebrates. This change of mind made tunicates less clear-cut invertebrates and shifted attention to other taxa as the key starting point for the invertebrate-to-vertebrate transition. Here again, Kowalevsky led the way [45], tentatively suggesting several phyla, including annelids, that might have been ancestral to vertebrates. His speculations were influenced by the earlier, non-evolutionary idea of Geoffroy-Saint Hilaire that the unity of body plans among animals is illustrated by the inverse dorsoventral arrangement of the main organ systems of vertebrates on the one hand and of annelids and arthropods on the other [46]. More specifically, Kowalevsky proposed that the notochord near the dorsal side of a vertebrate might have a homolog in the form of a band of fibrous cells (of unspecified nature) running in close association with the nerve cord near the ventral side of an annelid [45].

In 1875, Dohrn [47] and Semper [48] presented more extensive scenarios indicating how annelid-like ancestors underwent dorsoventral inversion while evolving into vertebrates. Both proposals were influenced by Kowalevsky’s suggestion that annelids had a fibrous notochord-like structure closely associated with the nerve cord. According to Dohrn, these fibers were muscles that emigrated away from the nerve cord and later transformed themselves into cartilage (unfortunately he included no illustrations). Semper [48] simply appealed to the authority of Kowalevsky that the vertebrate notochord could be traced back in evolution to fibers (of unspecified histological identity) associated with the annelid nerve cord. Semper provided illustrations of the annelid and vertebrate conditions (Fig. 2f, g), but ignored the intermediate stages.

Several contemporaries of Dohrn and Semper agreed with most of the annelid scenario but differed about how the notochord originated. Lwoff [49] proposed that annelids had a notochord that was cartilaginous from the beginning and never passed through fibrous stages, while Ehlers [50] suggested that that it arose from a siphon. The siphon is a tubular gut region, present in a few annelids and several other invertebrates, that opens at either end into the main course of the digestive tract and runs parallel and just ventral to the latter. Thus, a dorsoventral inversion of the annelid body (Fig. 2h) would orient the siphon in a position comparable that of the chordate notochord. To attain the vertebrate condition (Fig. 2i), the siphon detached from the rest of the gut and was converted from an epithelial tube to a compact cartilaginous structure. Two of Ehler’s contemporaries [51, 52] endorsed his siphon-to-notochord transition. Thereafter, however, with rare exceptions [53], the annelid scenario remained unpopular during much of the twentieth century. It was only after the passage of many years that advances in developmental genetics stimulated the present revival of the theory (to be examined in detail in a later section of this review).

### The nemertean theory

In 1883, Hubrecht [54] made the next attempt to turn invertebrates into vertebrates. He chose nemertean-like ancestors (not requiring dorsoventral inversion) to begin his scenario. The nemertean gastrula (Fig. 2j) has two invaginations, one becoming the archenteron and the other becoming the proboscis; at the same stage, numerous mesenchyme cells immigrate into the blastocoel. Subsequently, a through gut forms, and mesenchyme cells condense around the proboscis (Fig. 2k). Schizocoely then produces a proboscis sheath and proboscis coelom containing a few residual mesenchyme cells (Fig. 2l). Hubrecht’s conversion of a nemertian into a protovertebrate begins as the proboscis withdraws anteriorly from the proboscis coelom (Fig. 2m, arrow). The transition is completed when the mesenchyme cells in the proboscis coelom greatly multiply and form a cartilaginous notochord. At the same time, the remnant of the proboscis becomes the anterior hypophysis, while the two main lateral nerves migrate dorsally and fuse to form the dorsal nerve cord (Fig. 2n). Twentieth century revisions of the nemertean hypothesis [55] all retained Hubrecht’s way of making the vertebrate notochord. Since then, however, all versions of the nemertean scenario have been rendered highly improbable due to robustly supported molecular phylogenies relegating nemerteans to the Lophoptrochozoa, at a considerable phylogenetic distance from the chordates [56].

### The original enteropneust scenario

**Fig. 3:**
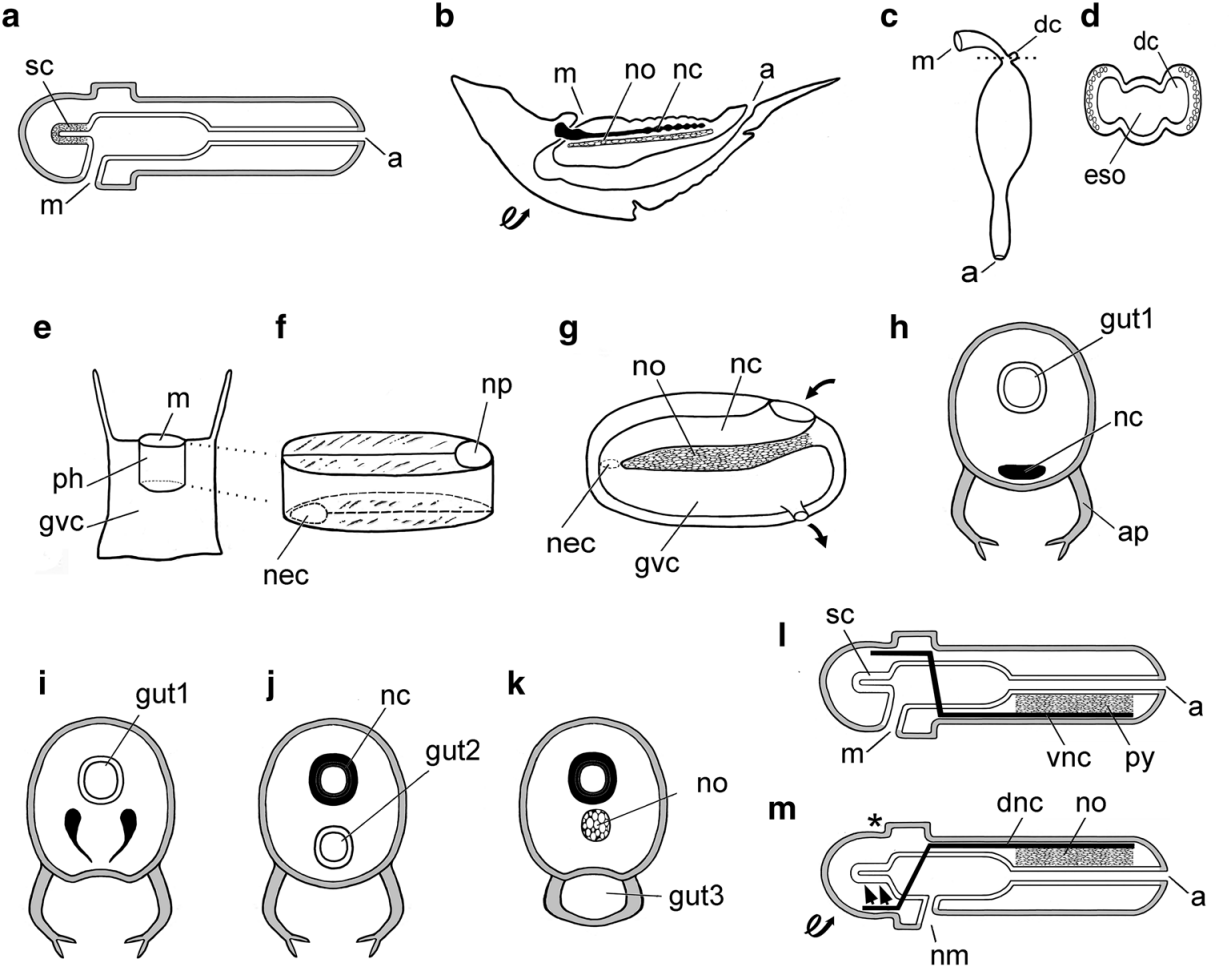
Notochords in phylogeny: enteropneusts, arthropods, phoronids, and cnidarians. **a** Enteropneust scenario (after Bateson [57]); the notochord homolog is the stomochord (sc); *m* mouth; *a* anus. **b** The inverted arthropod theory (after Patten [62]) with the notochord (no) ventral to the nerve cord (nc); *m* mouth; *a* anus. **c**, **d** The diplochord hypothesis (after Masterman [64]); **c** shows only the digestive system of the actinotroch larva (*m* mouth; *a* anus) with a pair of diplochords (dc). The cross section **d** (at the level of the *dotted line* in **c**) shows a diplochord (dc) on either side of the esophagus (eso). **e**–**g** Cnidarian hypothesis (after Lameere [69]). The sea anemone-like ancestor shown in side view (**e**) has an oblong mouth (m) leading to a pharynx (ph) and gastrovascular cavity (gvc); **f** shows the pharyngeal region mostly closing along the *top* and along the *bottom* to leave open, respectively, the neuropore (np) and the neurenteric canal (nec); in **g**, the notochord (no) has formed in the mesoglea between the nerve cord (nc) and the gastrovascular (gvc); *arrows* indicate the entry and exit of water. **h**–**k** Right-side up arthropod theory (after Gaskell [74]), starting with an arthropod (**h**) with appendages (ap), a primary gut (gut1), and a ventral nerve cord (nc); the nerve cord tissue migrates dorsally (**i**) and surrounds the primary gut (in **j**) to make a hollow, dorsal nerve cord (nc), while a secondary gut (gut2) forms by invagination from the ventral midline; subsequently (**k**), the secondary gut becomes the notochord (no) and a tertiary gut (gut3) is formed by the appendages fusing their tips. **l**, **m** Conversion of an arthropod-like enteropneust into a chordate (after Nübler-Jung and Arendt [87]). Before dorsoventral inversion (**l**), stomochord (sc) is not considered to be a notochord; the nervous system (in *black*) is annelid-like and includes a ventral nerve cord (vnc); in addition, a well-developed midventral mesentery, the pygochord (py) connects the gut to the ventral body wall; *m* mouth; *a* anus. After inversion (**m**, *looped arrow*), the anatomy becomes chordate-like as the original mouth disappears (*asterisk*), a new mouth (nm) opens and the old subesophageal ganglion migrates dorsally (*arrows*). In addition, the pygochord becomes the notochord (no) underlying the dorsal nerve cord (dnc)

Although earlier biologists had vaguely suggested that enteropneust hemichordates might be precursors of the vertebrates, Bateson, in 1886, was the first to present a detailed scenario for the conversion [57]. No dorsoventral inversion of the body axis was required (Fig. 3a). The notochord homolog was the stomochord, a short diverticulum projecting anteriorly from the buccal cavity and acting as a fulcrum to facilitate undulatory swimming. Disconcertingly, however, the hemichordate stomochord is regionally restricted and lacks any intimate association with the locomotory musculature [58]. During much of the twentieth century, Bateson’s scenario was occasionally revived, in whole or in part (for instance, Garstang appropriated the stomochord-to-notochord conversion [59]), although by no means broadly accepted. More recently, however, advances in developmental genetics have led to modern versions of the enteropneust theory, as will be discussed in a subsequent section of this review.

### Further classical scenarios: arthropod, phoronid, and cnidarian notochords

In 1890, Patten published an extensive scenario for deriving vertebrates from inverted arthropods [60]. At the time, arthropods and annelids were considered to be close relatives—incorrectly, it later turned out [61] —so Patten’s scenario bears a general resemblance to the annelid theory. He derived the notochord from a medial strand of tissue associated with the forming nerve cord. The strand then hollowed out and temporarily formed a spinal artery that transitioned to a notochord when its lumen filled with vesicular cells (Fig. 3b). He reiterated his earlier ideas in a 1912 book [62] that had an almost universally negative reception [63], and his work has been largely ignored ever since.

In 1897, Masterman proposed that phoronid larvae (called actinotrochs) had notochord-like structures [64] in the form of two small diverticula arising on either side of the developing esophagus (Fig. 3c, d). These diverticula, which he termed diplochords, although not medially located, reminded him of the hemichordate stomochord. He thus concluded that diplochords were homologs of the vertebrate notochord. Subsequently, Kemna [65] accepted the diplochord idea, while Roule [66] modified it slightly by proposing that the evolutionary precursor of the notochord was an unpaired diverticulum arising from the larval foregut of phoronids. Although adult phoronids lack a notochord, Masterman’s proposed notochord homologs were given credence during most of the twentieth century because they fit well with other morphologic features seeming to unite phoronids with deuterostomes [67]. More recently, however, molecular phylogenetic analysis has convincingly moved the phoronids from the deuterostomes to the lophotrochozoans [68], and the putative notochordal nature of the diverticula of phoronid larvae is all but forgotten.

In 1905, Lameere [69] published a scheme for notochord evolution that started with cnidarians—specifically with a creature resembling a sea anemone. A modern sea anemone has an oblong mouth opening into a sleeve-shaped pharynx projecting downward (aborally) into the gastrovascular cavity (Fig. 3e). Lameere commenced his scenario by zipping the sea anemone mouth shut until only a small opening remained at one end; simultaneously, he zipped the aboral exit of the pharynx shut in the opposite direction, leaving only a small opening (neurenteric canal) leading to the gastrovascular cavity (Fig. 3f), thereby converting the pharynx into a hollow dorsal nerve cord with a neuropore (functioning as small mouth through which water and food particles enter the animal). As already mentioned, the opening between the nerve cord and gastrovascular cavity became a neurenteric canal, permitting water to enter the gastrovascular cavity. To account for water outflow, Lameere surmised that an exit pore (comparable to the club-shaped gland of amphioxus) opened on the right antero-ventral side of the body (Fig. 3g). At this stage, the creature was still only a very early chordate comparable to an amphioxus larva without mouth, anus, or gill slits. A notochord then originated from an accumulation of cartilage-like cells (Fig. 3g) in the mesogloea between the nerve cord and the gastrovascular cavity. Eventually, the vertebrate condition was attained when a mouth, anus, and gill slits developed to connect the cavity of the gastrovascular cavity with the surrounding water.

The last of the classical scenarios for notochord evolution was proposed by Gaskell, remembered today mainly for his fundamental discoveries in physiology [70]. In the late 1880s, Gaskell had to give up his laboratory work to care for his chronically ill wife [71]. To keep himself intellectually engaged at home, he began developing a right-side up arthropod scenario for the origin of the vertebrates [72], eventually adding his ideas about notochord evolution [73]. He starts with an uninvited arthropod (Fig. 3h) in which the ventral nerve cord tissues migrate to (Fig. 3i) and surround the digestive tract, thus converting it to a hollow dorsal nerve cord while a secondary gut invaginators from the ventral surface of the body (Fig. 3j). Later, the secondary gut is filled with solid tissue to become the notochord while the tips of the paired appendages all along the body fuse in the ventral midline to enclose the definitive (tertiary) gut (Fig. 3k). In 1908, Gaskell’s book [74] summarizing his ideas was not well received. One reviewer [75] wrote “The momentous problem of vertebrate beginnings is still ‘on the knees of the gods.’ We gravely doubt whether Gaskell’s book will be of great value in dislodging it.”

### Notochords when big-picture phylogeny was out of style: World War I to 1960s

During much of the twentieth century, biologists were mainly concerned with evolution at relatively low taxonomic levels, and big-picture phylogeny was rarely considered. One exception was Gislén, who thought that the notochord had its beginnings in Palaeozoic echinoderms that ultimately evolved into vertebrates [76]. He began his scenario with the larval stage of an ancient echinoderm and proposed that the middle of the three coeloms on the left side (the hydrocele) was the precursor of the vertebrate notochord. His complex chain of reasoning, which we will not develop here, featured a bizarre amphioxus-like intermediate that ate with its neuropore and defecated through its mouth.

Following Gislén [76], several biologists have returned to the idea that the echinoderm hydrocoel might be a homolog of the vertebrate notochord [77, 78]. Until recently, such a coelomic origin seemed to be supported by the structure of the fully developed notochord in the tail of many tunicates—namely, an elongated epithelial bag surrounding a fluid-filled lumen [42]. Now, however, molecular phylogenies have rearranged the major taxa of chordates to locate amphioxus as the sister group to tunicates plus vertebrates [79]. Because neither amphioxus nor vertebrates have a coelom-like notochord at any stage of development [80], it is likely that the coelom-like arrangement is a peculiarity of tunicates and not a fundamental chordate property. This, in turn, makes it unlikely that echinoderms ever had a homolog of the notochord. For the sake of completeness, it should be mentioned that Jefferies [81] reconstructed a notochord running down the axis of what he considered to be the tail of fossils that he termed calcichordates. In contrast, more recent opinion [82] considers that such fossils represent echinoderms that had elements of the water vascular system occupying the position of the previously proposed notochord.

### The beginnings of molecular phylogeny and developmental genetics and revival of interest in notochord evolution

During middle decades of the twentieth century, most evolutionary biologists were concerned only with small-scale evolution, and most developmental biologists were not interested in evolution at any scale. In spite of this, the latter gave particular attention to the notochord—as a derivative of some cells of the primary organizer and as a source of substances influencing neighboring tissues [83]. In that era, frustratingly little progress was made in understanding these phenomena because the techniques for characterizing and manipulating minute amounts of nucleic acids and proteins were not available. By the 1960s, however, the situation began to improve. Evolutionary biologists took a new interest in big-picture phylogeny after Zuckerkandl and Pauling introduced molecular sequence-based tree construction [84] and, two decades later, the discovery of the homeobox [85] reinterested developmental biologists in evolutionary issues and the field of developmental evolutionary biology (devo-evo) emerged.

In 1994, Nübler-Jung and Arendt published the first devo-evo paper concerned, in part, with notochord evolution [86]. Then, in 1996, they followed with a more detailed scenario that began with an annelid-like ancestor that transitioned, via an enteropneust-like intermediate, to a chordate [87]. They argued on the basis of neuroanatomy and developmental genetics that the Dohrn/Semper annelid theory was preferable to Bateson’s enteropneust theory. Their key idea was that the nervous system of enteropneust-like ancestors of the chordates was arranged like that of annelids and arthropods (Fig. 3l); thus, dorsoventral inversion of the body would result in a chordate-like nervous system [87, 88]. As a corollary, they denied Bateson’s stomochord/notochord homology, and proposed instead that the notochord evolved at the enteropneust stage from the pygochord (Fig. 3l, m), a conspicuous midventral mesentery originally discovered by Spengel [89] and named by Willey [90]. However, the pygochord appears to be a peculiarity of only a few enteropneust species [91], and its homology with the chordate notochord has never been widely accepted.

### The revived annelid scenario and the axochord hypothesis

Arendt carried forward the work started with Nübler-Jung, using molecular genetic methods to gain insights into the invertebrate-to-vertebrate transition. Among other things, he compared such developmental genetic features as regionalization of *Hox* genes [92] and nerve cell specification [93, 94] between annelids and chordates. His results led him to reject his initial suggestion of an enteropneust bridge between annelids and chordates [87] and to propose instead a revived version of the Dohrn/Semper annelid scenario. To account for the origin of the notochord, Arendt and his colleagues began with an annelid-like creature that had a midventral longitudinal muscle closely associated with the nerve cord. They termed this muscle the axochord and proposed that it is the evolutionary precursor of the chordate notochord [95]. The proposed homology is mainly based on the involvement of similar transcription factors (brachyury, foxA, foxD, twist, soxD, and soxE) and signaling molecules (noggin and hedgehog) in the formation of both structures. In response to this proposal, Hejnol and Lowe [96] cautioned that the two structures may be homoplasies and not homologies, partly because most of the genes they express in common are involved in the development other tissues as well. The homology has also been questioned [97] because there is no evidence that the axochord synthesizes signaling proteins comparable to those produced by amphioxus and vertebrate notochords. In response to such criticisms, Arendt and others are expanding their search for possible axochord-like structures in a wide spectrum of invertebrate phyla [98]. It has even been suggested that an axochord-like muscle should be sought in enteropneusts [99], although the older literature does not mention any likely candidate structures running in either the dorsal or the ventral midline of the trunk [100].

### The revived enteropneust scenario and notochords as chordate novelties

Advances in molecular development not only led to a revived annelid theory, they also prompted Lowe to propose a new (and much modified) version of the enteropneust theory [101, 102]. In the original enteropneust scenario [57], Bateson postulated that the ancestor of the vertebrates already had many vertebrate-like features, including a notochord homolog. In contrast, Lowe proposed an enteropneust-like ancestor with a relatively uncomplicated morphology, lacking, among other things, a central nervous system. Surprisingly, in spite of their seeming morphological simplicity, enteropneusts proved to have an ectoderm divided into complex gene expression domains markedly congruent with those in the central nervous system of developing chordates. This led to proposals about how the simple tissues of an enteropneust-like ancestor could give rise to the complex organ systems of chordates; here, however, we will focus only on what is relevant for notochord evolution. In the view of Lowe and his associates, enteropneusts lack any structure homologous to the chordate notochord. Instead, they proposed that the “true” notochord evolved de novo in a basal chordate. They further speculated that this novel structure arose from cells running along the mid-dorsal side of the archenteron that took over and accentuated some of the signaling functions from ancestral endoderm and ectoderm [97].

More recently, a basic tenet of the revived enteropneust scenario—namely, the proposed morphological simplicity of the ancestor of the chordates—has required some revision. For example, it appears that enteropneusts actually do have a central nervous system [103, 104]. In addition, there has even been an indication that the stomochord might, after all, be homologous to the vertebrate notochord because both are the source of hedgehog signaling that could conceivably influence differentiation in the central nervous system [105]. However, this possible stomochord/notochord homology was not well supported when further work [106] failed to demonstrate the expression of additional enteropneust genes homologous to those involved upstream and downstream from notochord formation in chordates. In sum, the controversy over whether enteropneusts have any notochord precursors (either stomochord, or pygochord, or axochord) is far from a satisfactory conclusion.

Lowe and his colleagues were not the first to consider that the notochord is a chordate novelty. In 1955, Berrill proposed a scheme for the origin of the vertebrates [107] that resembled the older ideas of Garstang [59] in many respects—but not for the origin of the notochord. Whereas Garstang proposed the structure was derived from the hemichordate stomochord, Berrill thought that the notochord first appeared as a novelty in an ancestral tunicate larva [107]. The larva in question, which was originally tailless, suddenly acquired a notochord by a mutation that caused vacuolization along the roof of the archenteron. The new notochord pushed out a larval tail that became motile when axial muscles, also novelties, differentiated in the same body region.

Berrill’s focus on tunicates was understandable because, until recently, they were considered the basal chordate group. However, even after the rearrangement the chordates such that amphioxus is a sister to a tunicate plus vertebrate clade [79], tunicates remain important for elucidating notochord evolution [108]. In tunicates, as in other chordates, *brachyury* genes are required for many aspects of notochord development. Because tunicates are favorable material for working out gene regulatory networks, good progress has been made in elucidating how *brachyury* is involved in notochord development. Satoh et al. [109] have pointed out that, if one takes the position that the notochord is a chordate novelty, much of the problem of its evolutionary origin can be reduced to two questions. First, how did gene regulatory networks upstream from *brachyury* change to endow the gene, already involved in mesodermal differentiation of animals generally, with a new notochordal expression domain in chordates, and, second, what gene cascades link the expression of *brachyury* with the ultimate histodifferentiation of the definitive notochord? For ascidian tunicates, it is now possible to outline important reactions in the gene regulatory networks upstream and downstream from notochord-expressed *brachyury* [110]. Moreover, it is likely that homologous *brachyury*-related gene networks operate in other chordates [111], although such networks are as yet less well elucidated. Conversely, those who take the opposing position that the notochord originated deep within the tree of invertebrate life are also studying gene regulatory networks involved in the development of such structures as the annelid axochord [98] as possible support for their alternative scenario.

### Conclusions and perspectives

The evolutionary origin of the notochord remains an open question linked to the even broader uncertainty about the origin of the chordates [30]. From the current state of knowledge, it would be premature to decide whether the notochord is strictly limited to the chordates or is an ancient structure with homologs stretching back to a much earlier origin among the invertebrates. There has, however, been a useful advance that helps clarify thinking about notochord evolution: namely, the rearrangement of subphyla within the phylum Chordata such that amphioxus is now the sister group of the tunicate plus vertebrate clade [79]. The new arrangement makes it easier to accept tunicate features as derived instead of basal within the chordates. At the genetic level, tunicates are quite remarkable—they are characterized by very rapid evolution, a lack of synteny with other chordates, the organization of many coding sequences into operons, and a strong tendency to discard key developmental genes [112]. In the light of all these peculiarities, it is surprising that tunicates have retained such a clearly chordate-like phenotype [113] (this emphasizes how poorly we still understand genotype/phenotype relationships in general). In contrast, the developmental genetics of amphioxus and vertebrates are broadly similar (Table 2). Thus, it is reasonable to assume that, in comparison to tunicates, amphioxus might give a more accurate idea of the early history of the chordate notochord. Progress toward deciding between the current conflicting scenarios for notochord evolution will probably require a detailed knowledge of gene regulatory networks in a wide spectrum of animals, which will not be accomplished quickly. For the present, a more practicable approach could be more thorough studies of all aspects of the biology of enteropneusts, animals that figure large in chordate origin scenarios.

## Abbreviations

Fox: forkhead box
sox: sex determining region Y related box
